# Optimized isolation method of humin fraction from mineral soil material

**DOI:** 10.1007/s10653-021-01037-3

**Published:** 2021-07-16

**Authors:** Jerzy Weber, Elżbieta Jamroz, Andrzej Kocowicz, Magdalena Debicka, Jakub Bekier, Irmina Ćwieląg-Piasecka, Aleksandra Ukalska-Jaruga, Lilla Mielnik, Romualda Bejger, Maria Jerzykiewicz

**Affiliations:** 1 Institute of Soil Science and Environmental Protection, Wroclaw University of Environmental and Life Sciences, ul. Grunwaldzka 53, 50-357 Wrocław, Poland; 2 Institute of Soil Science and Plant Cultivation, Department of Soil Science, Erosion and Land Conservation, State Research Institute, ul. Czartoryskich 8, 24-100 Puławy, Poland; 3 Department of Bioengineering, Lab of Physics and Agrophysics, West Pomeranian University of Technology, ul. Papieża Pawła VI 3, 71-459 Szczecin, Poland; 4 Faculty of Chemistry, Biomaterials Chemistry Group, University of Wroclaw, ul. F. Joliot-Curie 14, 50-383 Wrocław, Poland

**Keywords:** Soil, Humic substances, Humin, Isolation, Extraction

## Abstract

Humic substances, including humin fraction, play a key role in the fate of organic and inorganic xenobiotics contaminating the environment. Humin is an important fraction of humic substances, which has been the least studied to date. This is due to the difficulties connected with its isolation that pose a number of methodological problems. Methods of humin fraction isolation can be divided into following main groups: (1) digestion of mineral soil components with HF/HCl followed by alkali extraction of HA and FA; (2) alkali extraction of HA and FA followed by extraction of humin by different organic solvents; and (3) alkali extraction of HA and FA followed by HF/HCl digestion of mineral soil components. Nevertheless, each of these methods has different limitations. We described in detail a useful procedure of humin isolation, in which this fraction was not extracted, but isolated from the soil by removing its soluble organic and mineral components. A modified method of HA and FA extraction with 0.1 M NaOH, according to the International Humic Substances Society, was used in the first step. Then, the mineral components in the residue were digested with the 10% HF/HCl. Unlike the procedures oriented to increase the concentration of organic matter, samples were treated several times with the HF/HCl mixture until the mineral fraction was almost completely digested. The main assumption of the method modification was to obtain the highest yield with the lowest possible ash content, but without affecting humin chemical structure. The results showed that the proposed procedure is characterized by a high efficiency and recovery and, therefore, it can be used to isolate high amounts of humin from soil.

## Introduction

Contaminants entering the soil can be accumulated in the upper soil horizon—from where they can be taken up by plants—or they can be moved to deeper genetic horizons as well as to groundwater. Their fate depends on the soil properties, especially the quantity and quality of soil organic matter (SOM), a complex mixture of chemically and physically nonhomogeneous organic compounds derived from decomposition of plant and animal remnants as well as macromolecular colloidal products of transformation of these constituents. The role of SOM, and especially humic substances (HS), is crucial in affecting most of the processes occurring in the soil environment and determining microbiological, chemical, and physical properties of the soil (Frąc et al., 2017; Jamroz, 2012; Jamroz et al., 2014; Olk et al., 2019a, b; Weber et al., 2018). SOM is the main component of the soil sorption complex, which determines the ability to adsorb heavy metals and both inorganic and organic xenobiotics. Therefore, the quantity and properties of SOM, among which HS play a key role in determining xenobiotics fate, are an important element of any study on soil contamination (Cwielag-Piasecka et al., 2018; Loffredo & Senesi, 2006).

Although HS research has been based on the analysis of substances extracted from soil with alkali for over 230 years (Achard, 1786), recently critical views on HS nature, isolation processes and specific properties have appeared in the scientific literature (Kleber & Lehmann, 2019; Lehmann & Kleber, 2015). Critics have claimed that alkali-extractable fractions are laboratory artifacts, hence unsuitable for studying natural organic matter structure and function in field conditions. This point of view is debatable and controversial, provoking the opposition of many specialists in SOM research (De Nobili, 2019; Hayes et al., 2017; Myneni, 2019; Olk et al., 2019a, b; Weber et al., 2018). The classical approach to HS research has strong support in the contemporary literature (Hayes & Swift, 2020) and is strongly supported by the International Humic Substances Society (http://humic-substances.org/). HS research continues to be an important element of SOM research, which is reflected in current textbooks (Brady & Weil, 2008; Horwath, 2015; Tan, 2014).

According to the classic approach, humic substances have been classified into three fractions based on water solubility: 1) humic acid (HA) insoluble under acidic conditions (pH < 2) but soluble at higher pH; 2) fulvic acid (FA) soluble at all pH conditions; 3) humin—the fraction not soluble in water at any pH value (Kononova, 1966; Stevenson, 1994). Humin, a term proposed by Berzelius, (1839), refers to a fraction resistant to microbial activity, which usually constitutes about half of the HS in soil and more than 70% of that in lithified sediments, thus resulting the most abundant class of organic substances in the terrestrial environment. For many years, there was an agreement that among humic substances, HA and FA have the most important environmental significance. Recently, it is becoming apparent that the humin fraction plays an important role in the improvement of soil properties and in atmospheric carbon sequestration (Hayes et al., 2017).

After more than 180 years of research on humin, there is much less information on it than on any other HS fraction (Hayes et al., 2017). It forms very stable humic–clay complexes, which cannot be destructed during HS extraction (Stevenson, 1994). The lack of solubility and the recalcitrant chemical nature of this fraction makes it significantly difficult to study. Due to that, despite the fact that it is a key component of SOM, humin composition and properties have been rarely studied.

To isolate humin from soil material, different methods have been attempted (Hayes et al., 2017). They can be divided into following main groups: (1) digestion of mineral soil components with HF/HCl followed by alkali extraction of HA and FA (Tatzber et al., 2007; Zhang et al., 2017); (2) alkali extraction of HA and FA followed by extraction of humin by different organic solvents, as DMSO (Hayes, 2006) or MBIK (Rice and McCarthy 1989; Almendros et al., 1996); and (3) alkali extraction of HA and FA followed by HF/HCl digestion of mineral soil components (Kang & Xing, 2005; Li et al., 2015; Zhang & Katayama, 2012). Nevertheless, each of these methods has different limitations.

The advanced methods of studying the HS properties usually require the substance to be in solution; thus, an organic solvent for humin has been sought for many years. The MIBK (methylisobutylketone) method, introduced by Rice and MacCarthy, (1989) involves the partitioning of humin between an aqueous phase of varying pH and the MIBK layer. Almendros et al., (1996) used ultrasonic disaggregation followed by flotation in a bromoform–ethanol mixture and then partitioning in water-MIBK. Spaccini et al., (2000, 2006) have extracted a hydrophobic humic fraction from soils by means of an acetone-HCl (8:2, v:v) solution. Dimethylsulfoxide (DMSO) acidified with HCl has been studied for the isolation of humin-type materials (Clapp & Hayes, 1996); however, Zhu et al., (2005) found that a DMSO solvent system isolated less than 22% of total humin materials from different soils. Work of Simpson et al., (2007) has compared the amounts of total organic matter isolated with DMSO-d6 (deuterated dimethylsulfoxide), containing small amounts of acids, such as trifluoroacetic acid (TFA) and D_2_SO_4_. Hayes et al., (2017) proposed exhaustive extraction with 0.1 M NaOH + 6 M urea, wash out the base/urea (or dialyze), dry, and then exhaustively extract with DMSO with sulfuric acid. Bearing in mind the controversy around the use of extraction to obtain HS individual fractions, we chose the method in which humin was not extracted, but directly isolated from the soil by removing soluble organic and mineral components.

The main objective of the research was the quantitative and qualitative isolation of humin according to a procedure that apparently does not change the structure of the components and is characterized by high efficiency/recovery. In this paper, we described in detail the procedure of humin isolation optimized to yield large amounts of humin.

## Materials and methods

We isolated the humin fraction from eight mollic horizons of Chernozems and Phaeozems derived from different parent materials in different regions of Poland (Table 1), thus indicating different properties (Table 2) and diverse agroecological conditions. Particle size distribution was analyzed by the sieve and hydrometric method (Pansu & Gautheyrou, 2006), following the pretreatment that involved removal of organic matter and chemical dispersion with sodium hexametaphosphate. Soil pH was measured potentiometrically in 1 M KCl suspensions at the ratio of 1:2.5, total organic carbon (TOC) and total nitrogen (TN) were determined by dry combustion (Vario MacroCube, Elementar), CaCO_3_ content was determined with Scheibler method, cation exchange capacity (CEC) was calculated as the sum of exchangeable base cations extracted by 1 M NH_4_Ac and soil acidity determined in 1 M KCl. All soils were sampled from typical arable lands due to the high exposure of these soils to the accumulation of pollutants and at the same time their risk of penetration into the food chain.

**Table 1:** Localization and general information on soils

Sample no	GPS coordinates	WRB soil group	Cultivated plant
1	N 51° 11′ 27,79″; E 17 ^o^ 02′ 08,24″	Gleyic/Stagnic Phaeozems	triticale
3	N 50° 34′ 30,50″; E 17 ^o^ 55′ 59,81″	Rendzic Phaeozems	maize
6	N 50° 59′ 00,04″; E 16 ^o^ 56′ 52,48″	Gleyic/Stagnic Phaeozems	maize
7	N 50° 49′ 11,87″; E 16 ^o^ 52′ 39,38″	Calcic/Haplic Chernozems	sugar beets
8	N 50° 40′ 53,98″; E 16 ^o^ 55′ 47,78″	Gleyic/Stagnic Phaeozems	maize
9	N 50° 43′ 32,91″; E 23 ^o^ 50′ 05,94″	Calcic/Haplic Chernozems	wheat
10	N 53° 09′ 57,87″; E 14 ^o^ 55′ 15,19″	Gleyic/Stagnic Phaeozems	sugar beets
11	N 54° 03′ 53,67″; E 21 ^o^ 21′ 09,66″	Gleyic/Stagnic Phaeozems	triticale

**Table 2:** Main properties of soils

Sample no	pH (KCl)	TOC	N	C/N	CaCO_3_ g kg^−1^	CEC cmol( +) kg^−1^	% particles > 0.002 mm	USDA textural class
		g kg^−1^						
1	7.71	13.3	1.06	12.5	1.46	28.3	16	sandy loam
3	7.45	24.4	2.14	11.4	3.43	50.0	41	clay
6	7.52	21.2	1.60	13.2	1.53	33.4	22	loam
7	5.64	41.7	3.39	12.3	0.51	53.2	24	silt loam
8	7.39	26.1	2.03	12.8	1.03	21.6	19	silt loam
9	7.52	39.9	2.90	13.7	3.26	52.5	21	silt loam
10	7.48	24.6	2.12	11.6	1.54	34.4	24	loam
11	6.66	37.7	2.80	13.4	0.61	25.8	47	clay

The proposed modified methodology of humin isolation comprises several related extraction steps. Thus, the first step based on the exhaustive extraction of HA and FA is according to IHSS method described by Swift, (1996); however, we introduced some modifications to obtain the highest possible recovery. In the second step, a coarse fraction of silica was mechanically removed from HA and FA free residue, and the remaining precipitate was digested with the HF/HCl mixture to remove the mineral components. Finally, the sample was purified and freeze-dried.

The C concentration in the supernatant was determined with the dichromate-oxidation Tiurin–Simakov-modified method (Mebius, 1960). Obtained humin yield and ash content were correlated with properties of the source soil material using Statistica 13 for Windows.

## Humin isolation procedure

### Sample preparation

Each soil sample was dried at room temperature, then the roots and other plant remnants were removed. The material was ground and sieved to pass a 2-mm sieve. To remove coarse fractions, the sample with high sand content (above 20%) was additionally sieved to pass 0.5-mm sieve. The sample weighing approximately 200 g was placed in a 1000 cm^3^ polypropylene centrifuge bottle (recommended Thermo Scientific Nalgene 1000 cm^3^ PPCO bottle, resistance to centrifugal force 7100 g). Carbonate-rich material was treated with 10% HCl as long as CO_2_ bubbles appeared, then left for the next day. If CO_2_ was still released, 10% HCl was added until the reaction vanished.

### Removal of the HA and FA fractions

After addition of approximately 100 cm^3^ of 1 M HCl, carbonate-free or decarbonated sample was mixed thoroughly with baguette, then the reaction was controlled with a litmus paper to check whether the pH was maintained between 1 and 2. If necessary, the sample was additionally acidified by adding a small amount of 1 M HCl. Then the bottle was filled with approximately 800 cm^3^ 0.1 M HCl up to the level of the cap. After that, the sample was shaken thoroughly, so that there was no soil material sticking to the bottom of the bottle, and then shaken for 1 h in the rotary shaker. After shaking, the sample was centrifuged at 1000 g for 10 min. Supernatant (yellow colored so-called light fulvic fraction) was discarded, and 25 cm^3^ of 1 M NaOH was added to the sediment. After mixing the contents thoroughly, the reaction was checked with a litmus paper to confirm the pH around 7. If necessary, a small amount of 1 M NaOH was added until the reaction was neutral.

The extraction of HS with NaOH extraction is a widely accepted method for SOM research. To reduce the risk of harmful changes in the structure of HS, 0.1 M NaOH is used because of its milder nature than 0.5 M NaOH. The soil: extractant w/v ratio of 1: 5 (Tan, 2014) or 1: 10 (IHSS method) is the most commonly used, although Kuwatsuka et al., (1992) indicated that the 1: 10 ratio is insufficient when the soil contains a large amount of organic matter. They suggested that the ratio of soil carbon to the extractant used may be the most decisive. We decided to adopt the soil: NaOH ratio of 1: 5; however, extraction was exhausting and therefore the ratio of NaOH to the soil carbon remaining in the residue increased with each extraction cycle. This enabled to design a faster procedure and to obtain the possible highest yield of humin.

After neutralization, the suspension was refilled with 0.1 M NaOH up to a volume of 1000 cm^3^, which made soil: NaOH w/v ratio of 1: 5 (about 1:10 v/v). The sample was shaken for at least 4 h. We tested the effect of different shaking times on the amount of extracted humic substances. To assess the differences, each supernatant was analyzed for the concentration of carbon, which corresponded to the amount of HA and FA extracted. Shaking for 20 h proved to be more effective (Table 3), but the advantage of 4-h shaking intervals offered the possibility of extracting twice a day. Due to that, a greater amount of HA and FA was removed daily. Then, the sample was centrifuged at 3000 g for 20 min. The samples with high content of colloidal fraction required centrifuging extended to 30 min. The dark-colored supernatant with HA and FA was discarded, and the sediment was again flooded with 0.1 M NaOH to a volume of 1000 cm^3^, thoroughly shaken so that no material sticks to the bottom or sides of the bottle, and shaken for at least 4 h. The extraction was repeated until the supernatant was almost colorless. Depending on the content of organic matter in the soil material, the procedure required from five up to a dozen extraction cycles.

**Table 3:** The concentration of C in the supernatant after different shaking time

Sample Nr	Concentration C (g cm^−3^)	
	4 h	20 h
1	0.29	0.29
3	0.32	0.44
6	0.59	0.89
7	1.37	2.15
8	1.08	1.06
9	0.75	0.82
10	0.35	0.76
11	1.37	1.78

### Removal of the mineral components

To remove mineral constituents from HA and FA free material, we used 10% HF/HCl (20% HF: 20% HCl = 1:1), which can effectively eliminate the mineral matrix, and concentrate organic matter (Kögel-Knabner, 1997; Rumpel et al., 1998; Schmidt et al., 1997). Although the HF/HCl treatment can lead to hydrolysis and loss of polysaccharide and protein materials (Stevenson, 1994; Wang & Xing, 2005), the advantage of using HF is the removal of metal ions, which facilitates the use of spectroscopic techniques to characterize humin. Unlike the procedures directed at increasing the concentration of organic matter (Schmidt et al., 1997; Zhang & Katayama, 2012), the sample was treated with the HF/HCl mixture until the mineral fraction not complexed with humin was completely dissolved. We tested mineral fraction dissolution in 10% HF/HCl solution using centrifuge bottles adopted to our procedure. Our tests showed that it takes 6 weeks to digest 200 g of pure sand in a 1000 cm^3^ bottle, with a weekly HF/HCl exchange. After replacing HF/HCl every 2 weeks, the time of digestion was extended to 8 weeks. Additionally, we compared the HF digestion of sand with the use of different shaking options. These tests showed that shaking is necessary for faster quartz dissolution, but shaking once a day is just as effective as continuous shaking.

After the last centrifugation with NaOH extraction, the samples were decanted, the supernatant was discarded, and the sediment was repeatedly flooded with minimal amounts (to the height of liquid about 1 cm above the sediment) of a mixture of 10% HF/HCl and thoroughly mixed. When using HF, special precautions were taken, as HF dissolves glass, penetrates latex, and is highly corrosive and acute toxic (Lippert et al., 2020). All actions were performed with nitrile gloves (latex gloves are not recommended) under a working fume hood. After tens of seconds, the suspension was gently decanted into a second bottle, so as not to transfer the heaviest and the thickest mineral fractions deposited at the bottom. After multiple washings, at the bottom of the bottle, a bright mineral fraction of sand with a few black grains of the so-called black carbon fraction remained, which was further discarded.

The bottle with the sample released from the coarse mineral fraction was replenished with 10% HF/HCl almost to full, shaken well, and left overnight. The next day, the bottle with the suspension was centrifuged at 3000 g for 5 min. Supernatant was discarded and the sediment was refilled with fresh 10% HF/HCl mixture. The humin suspension was left for a week. To ensure uniform HF/HCl penetration into the material, the bottle was shaken daily. After 6 days, the sample was centrifuged at 3000 g for 5 min, the supernatant was discarded, and the sample was refilled with HF/HCl mixture to make the bottle nearly full. The humin suspension was again left for a week, with shaking every day, as previously.

**Fig. 1:**
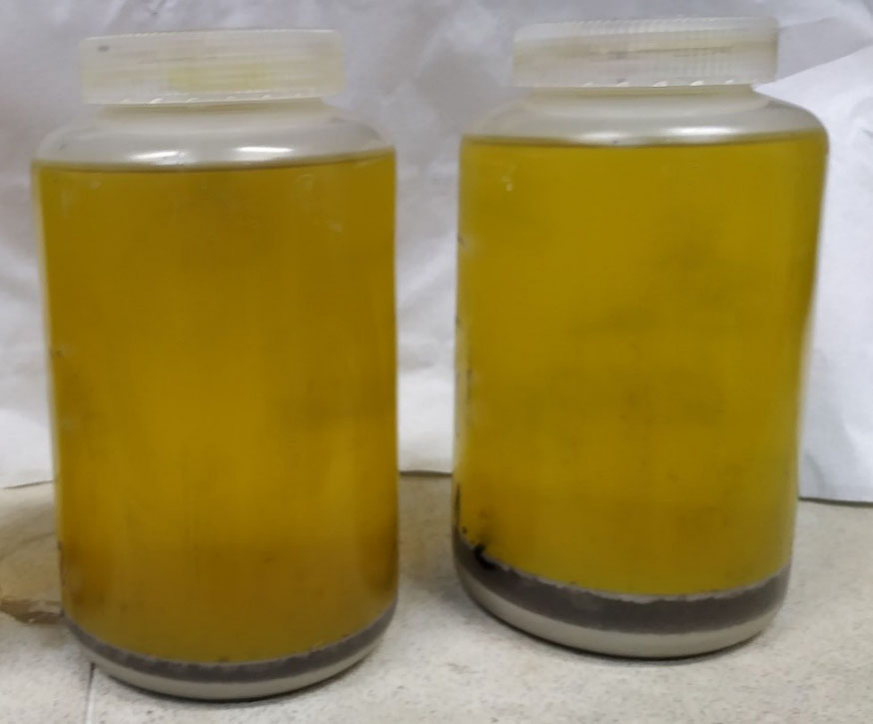
The delaminated sample after preliminary HF/HCl treatment. The upper darker layer is humin mixed with mineral part, while the lower-more coherent, white layer is silica

Depending on the material, after two or three weeks of mineral components removing, deposited in a bottle material delaminated into whiter and darker layers. Then the sample was centrifuged at 3000 g for 5 min, and the supernatant was discarded. Remaining sediment was refilled with a small amount (up to 1–2 cm height) of the HF/HCl and then mixed. After a tens of seconds, the sediment left delaminated into a darker layer (humin mixed with mineral part) and a deeper white layer (silica), which was more coherent (Fig. 1). This allowed the two layers to be separated by gentle mixing and decanting. The suspension with the darker part of the sediment was gently transferred into a new bottle so as not to disturb the deposited coherent white mineral fraction. The darker portion was collected for further procedure, while the remaining part was washed with little HF/HCl as long as the white cohesive layer was not disturbed. Then the sample was mixed with a small amount of HF/HCl and left for several tens of seconds to settle again and delaminate into white and darker layers. This separation was continued as long as a clearly white layer was observed. White mineral sediment was discarded, while the darker suspension was combined with the previously separated fraction and further treated with the HF/HCl mixture for 3–5 weeks until the precipitate was uniform in color (not differentiated into gray and darker layers).

### Final purification of humin and freeze-drying

The suspension was centrifuged at 3000 g for 5 min. The supernatant was discarded and humin remaining in the bottle was refilled with 10% HCl to half of the bottle volume, then left for 3 h to dissolve secondary minerals such as fluorite that could be formed during the extraction. After this time, the sample was centrifuged at 3000 g for 10 min, and the supernatant was discarded. Then distilled water was added to the humin residue, mixed, and left overnight. The next day, the sample was centrifuged at 3000 g for 10 min, and the supernatant was discarded. Distilled water was added again to the humin fraction and left for 3 h. This was repeated until a full neutralization of the supernatant. Then the sample was transferred to the dialysis tube and dialyzed against distilled H_2_O until Cl^−^ ions was no longer detected with AgNO_3_ and freeze-dried to get a powder of humin fraction.

## Summarizing remarks and conclusions

**Fig. 2:**
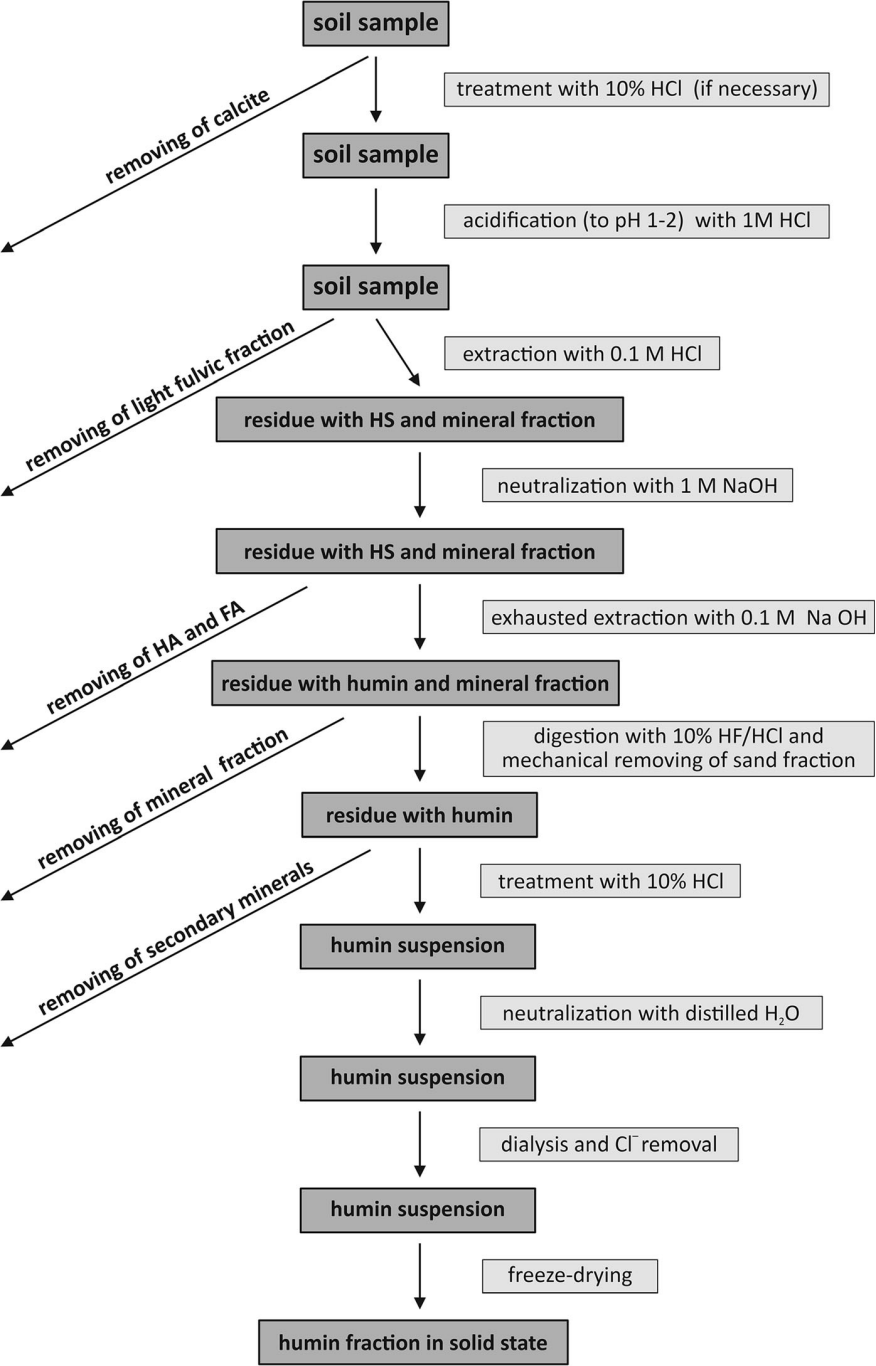
Simplified diagram of humin isolation

The presented procedure, which simplified diagram is shown in Fig. 2, enables to isolate a large amount of humin fraction from soil material in a possible short time. The whole isolation of humin takes about 9–10 weeks, depending on the SOM content. The extraction time of HA and FA clearly depended on the organic matter content. Soil containing higher amount of organic matter requires over a dozen of extraction cycles with NaOH, which can be completed after just a few cycles in case of the soil poor in clay and rich in sand fraction. When maximum shortening of the procedure is necessary, extraction with NaOH twice a day is recommended. If, on the other hand, time is not a limitation and one extraction of HA and FA is performed per day, extending the shaking time from 4 to 20 h increases its effectiveness (Table 3).

The procedure can be shortened at the stage of the HF/HCl digestion by mechanically removing the sand fractions after the first 2–3 weeks of the treatment. Reducing the treatment time shortens the contact of the sample with the HF/HCl mixture and limits the risk of the HF/HCl interference in the humin structure. The optimal HF/HCl digestion requires weekly replacement of the fresh mixture, and daily shaking. Performing it once a day is sufficient and as effective as continuous shaking. The HF/HCl digestion should be continued as long as the deposited material does not delaminate into darker and brighter layers, which is a symptom of incomplete digestion of mineral components.

During the isolation of humin, yield of humin ranged from 0,78 to 4.28% (Table 4), depending on the soil material. The highest humin yield was obtained from Calcic/Haplic Chernozems (samples 9 and 7) and some Gleyic/Stagnic Phaeozems (samples 10, 8 and 11). The humin content was correlated with the TOC content, while no correlations were found with other properties of the soil material (Table 5). Similarly, the ash content varied widely, from 22.89 to 54.50%, and showed no correlation with the properties of the soil (Table 4, 5). This indicates that the humin content in soil as well as ash content depend rather on the SOM properties and the environmental conditions of its accumulation than the properties of the soil material.

**Table 4:** The yield of humin isolated and ash content

Sample Nr	Weight of the soil for isolation (g)	Obtained amounts of humin		Ash (%)
		(g)	(%)	
1	10,040	78.8	0.78	39.45
3	6930	110.9	1.60	22.89
6	6880	90.7	1.32	38.57
7	5940	149.5	2.52	41.67
8	5190	107.1	2.06	28.01
9	2180	92.4	4.28	43.31
10	4650	123.7	2.66	54.50
11	5480	106.5	1.94	48.87

**Table 5:** Correlations between soil properties (pH, TOC, C/N ratio, CaCO_3_, clay content, CEC), yield of humin and its ash content

	pH	TOC	C/N	CaCO_3_	> 0.002 mm	CEC
Humin yield	− 0.151	0.732*	0.296	0.313	− 0.366	0.520
Ash content	− 0.193	0.279	0.219	− 0.377	− 0.034	− 0.093

^*^significant correlation coefficients

> 0.002-mm clay content

There are many different methods of humin isolation, including extraction with various organic solvents, all of them having particular limitations. The presented procedure seems to be the most optimal and reasonable way to isolate and purify large amounts of humin that may be used for further analyses. The humin extracted by this method constitutes the organo-mineral complexes representing the SOM fraction being the most strongly associated with the soil mineral fraction. Humin has a protective effect on mineral colloids, preventing them from an ultimate digesting in HF. Nevertheless, in the light of the criticism of studying the humic substances in the form of the soil extract, the investigation of humin isolated in solid state seems to be less controversial than the study of this fraction obtained by dissolution. It is even more important, since advance in the spectroscopic methods allow one to measure a substance in a form of a powder or in a paste.

## Data Availability

Statistica 13.3 for Windows, serial no. JPZ007B482801ARACD-9.

## Data availability

The authors confirm that complete raw dataset is included in the manuscript text and tables.
